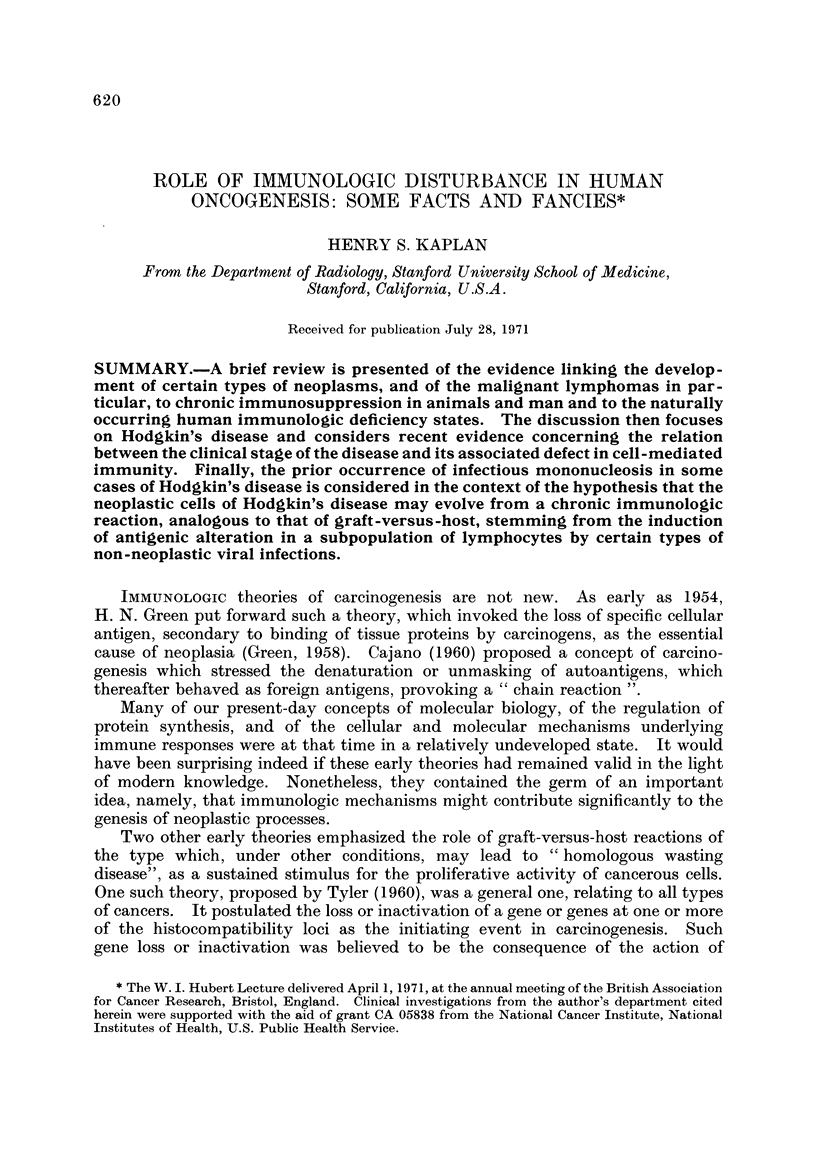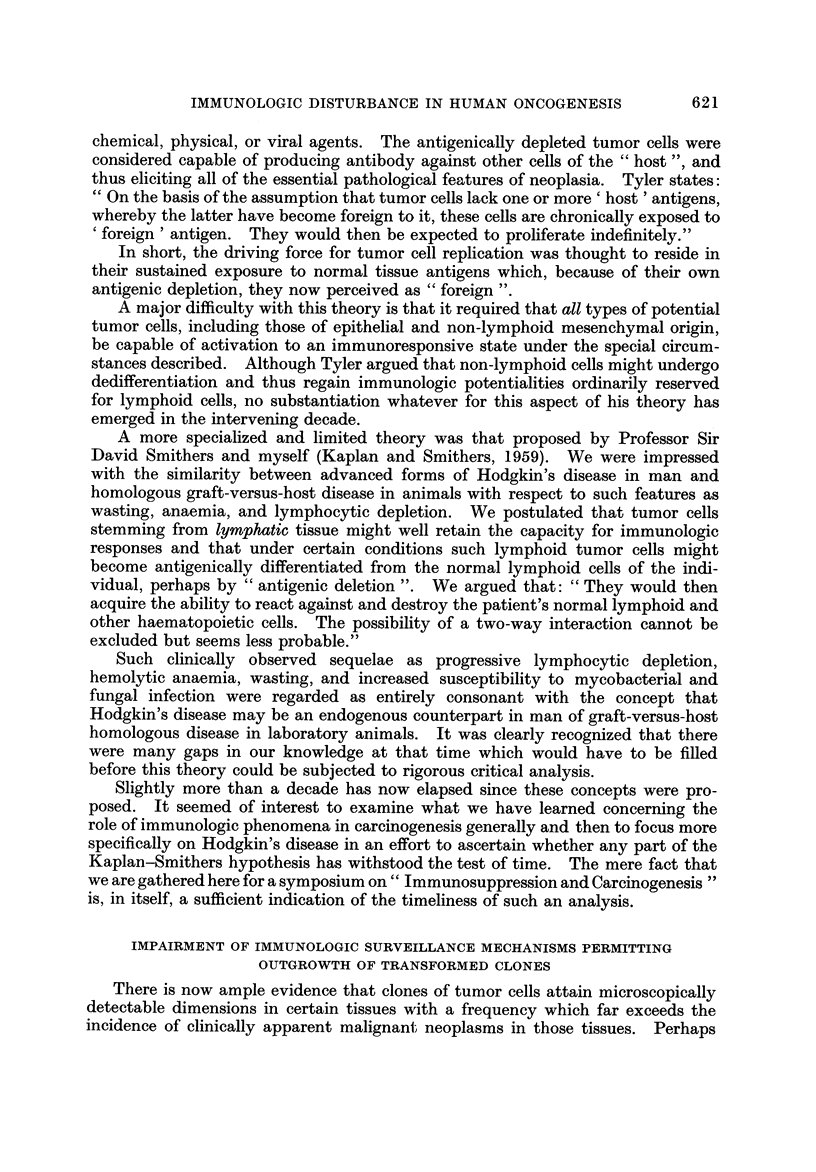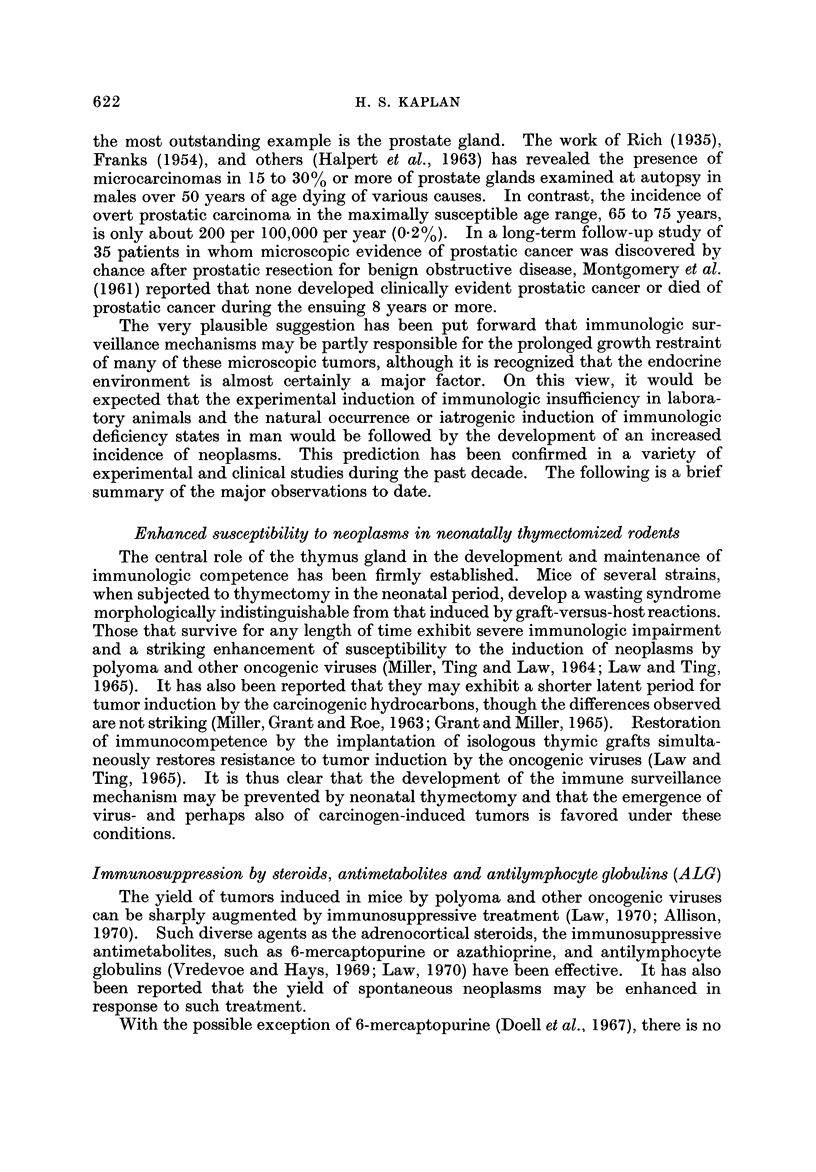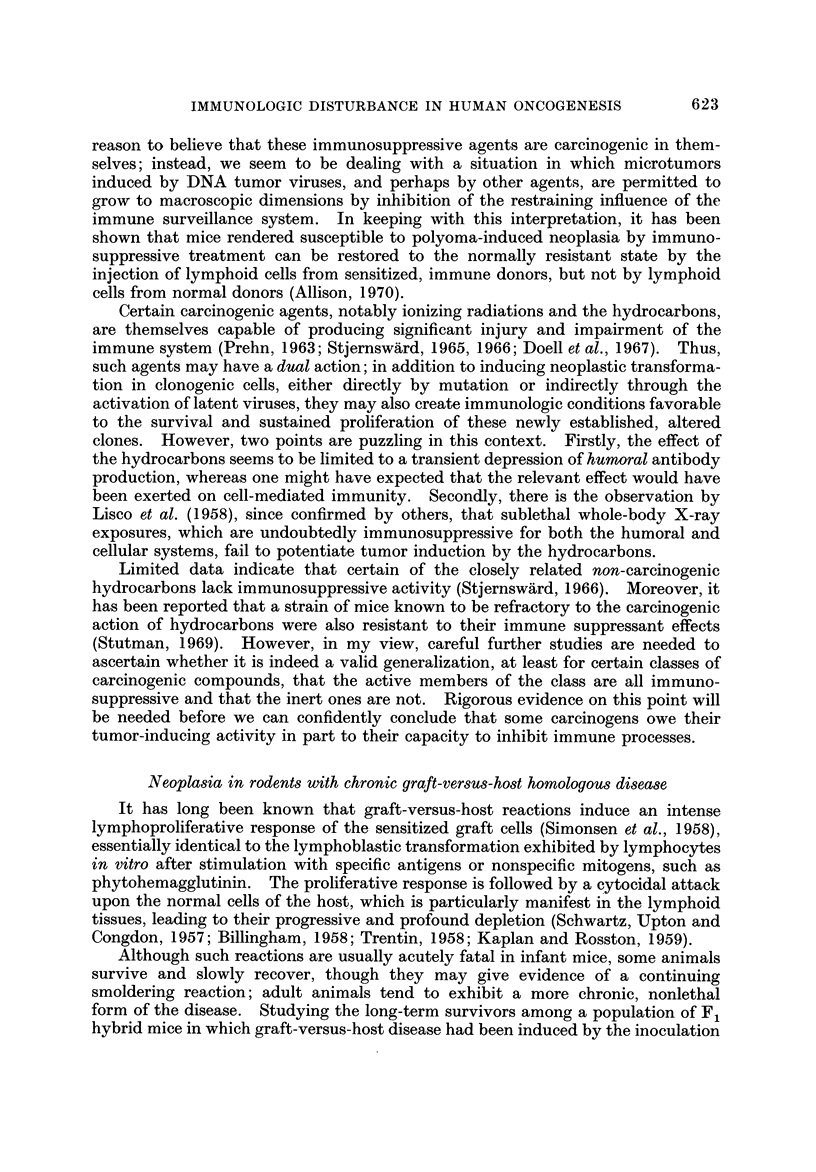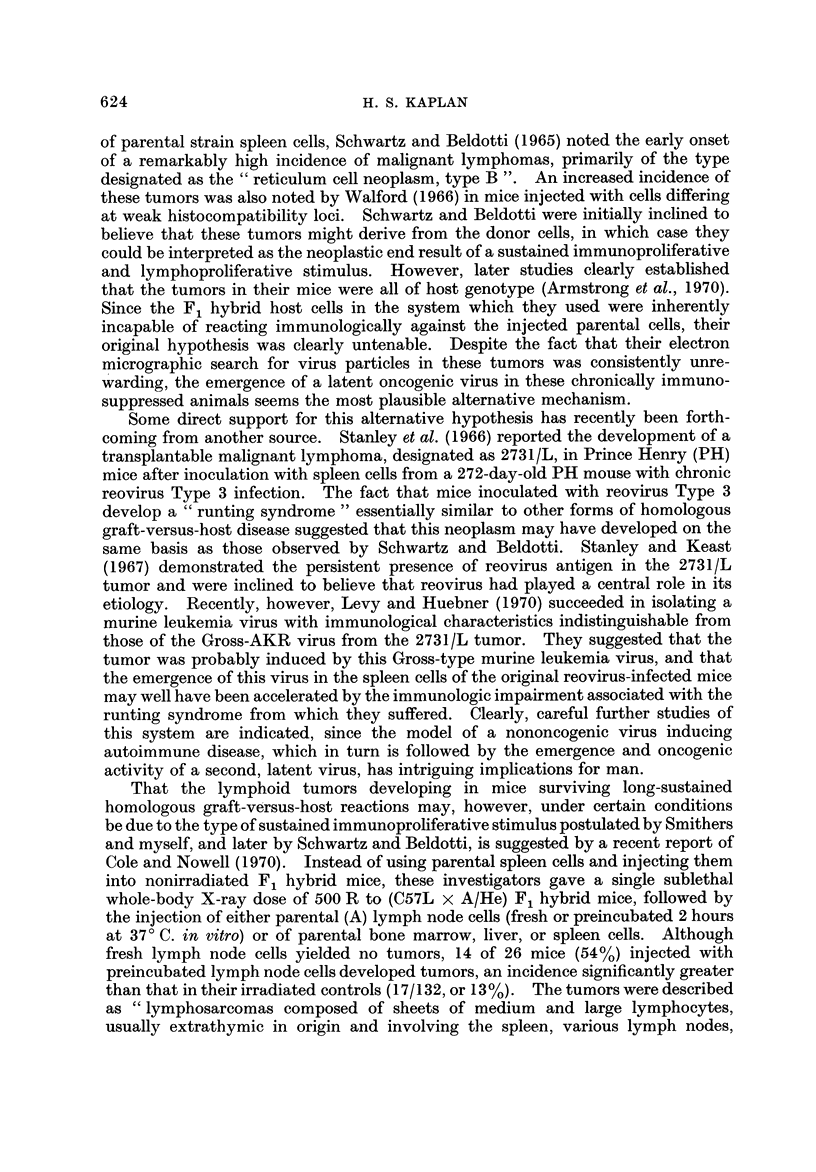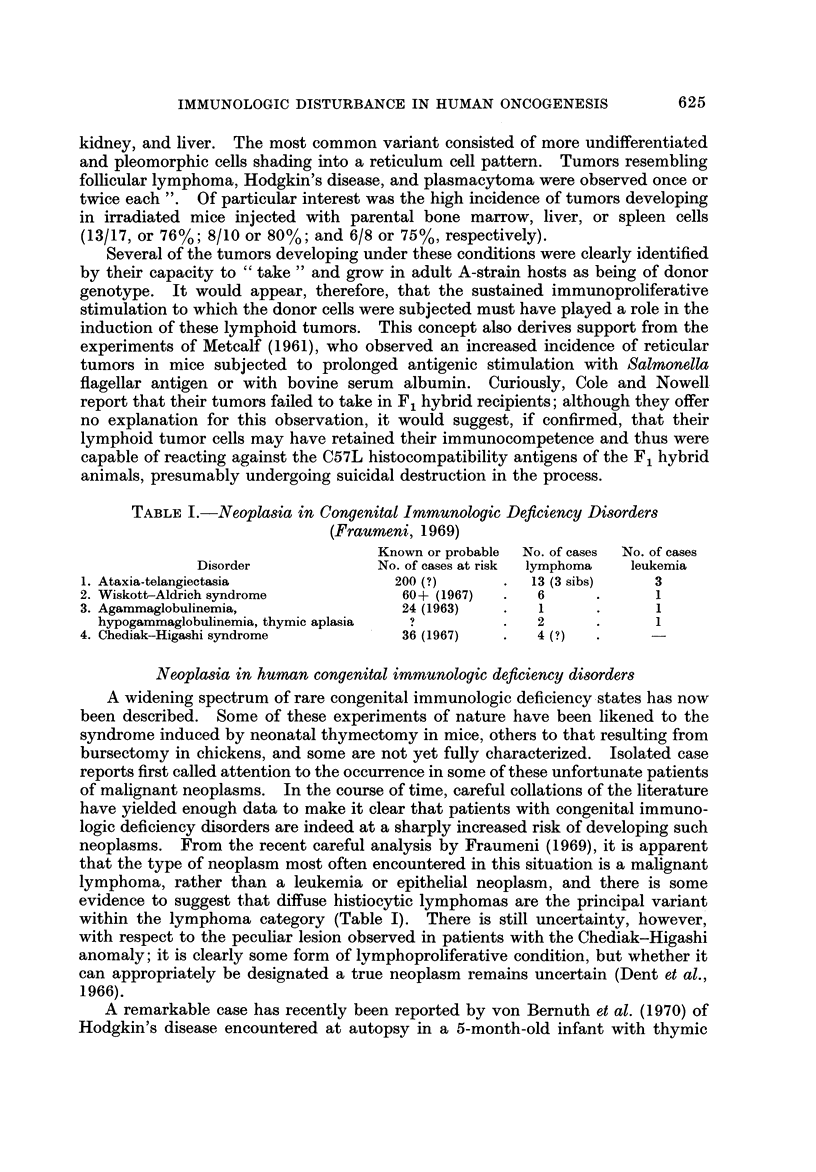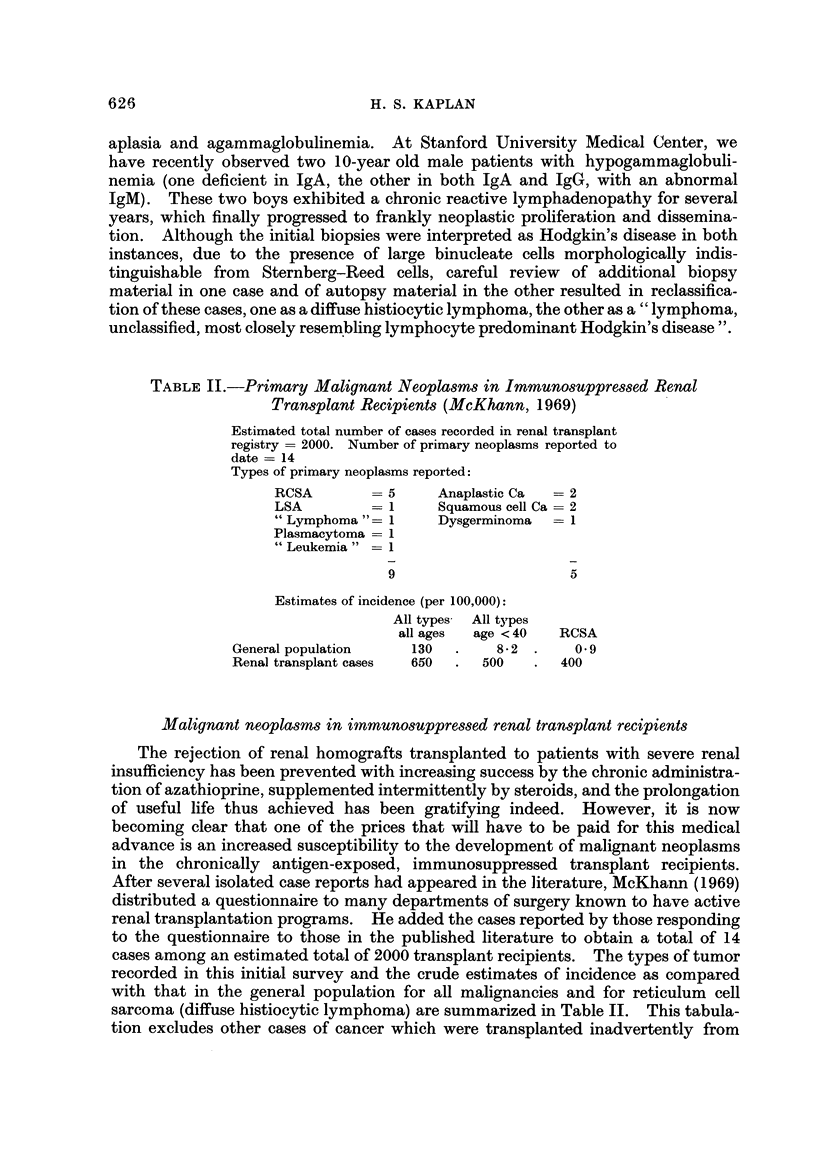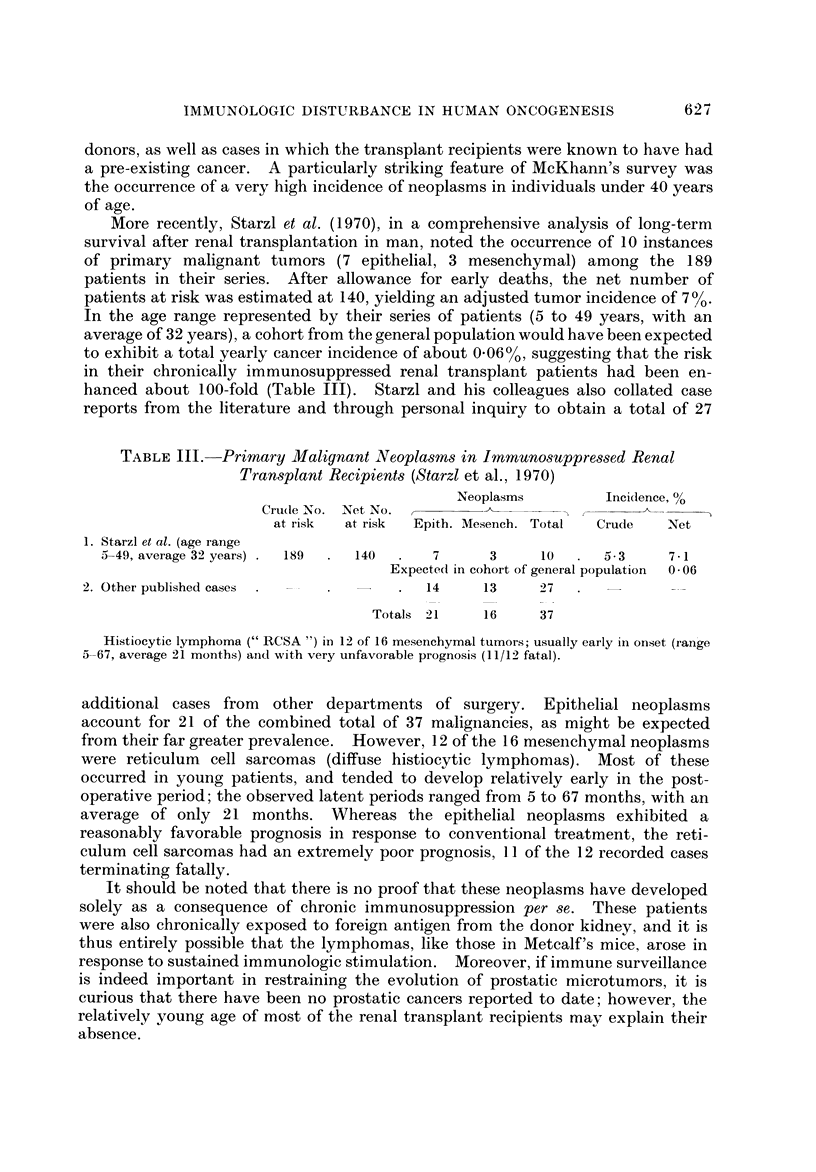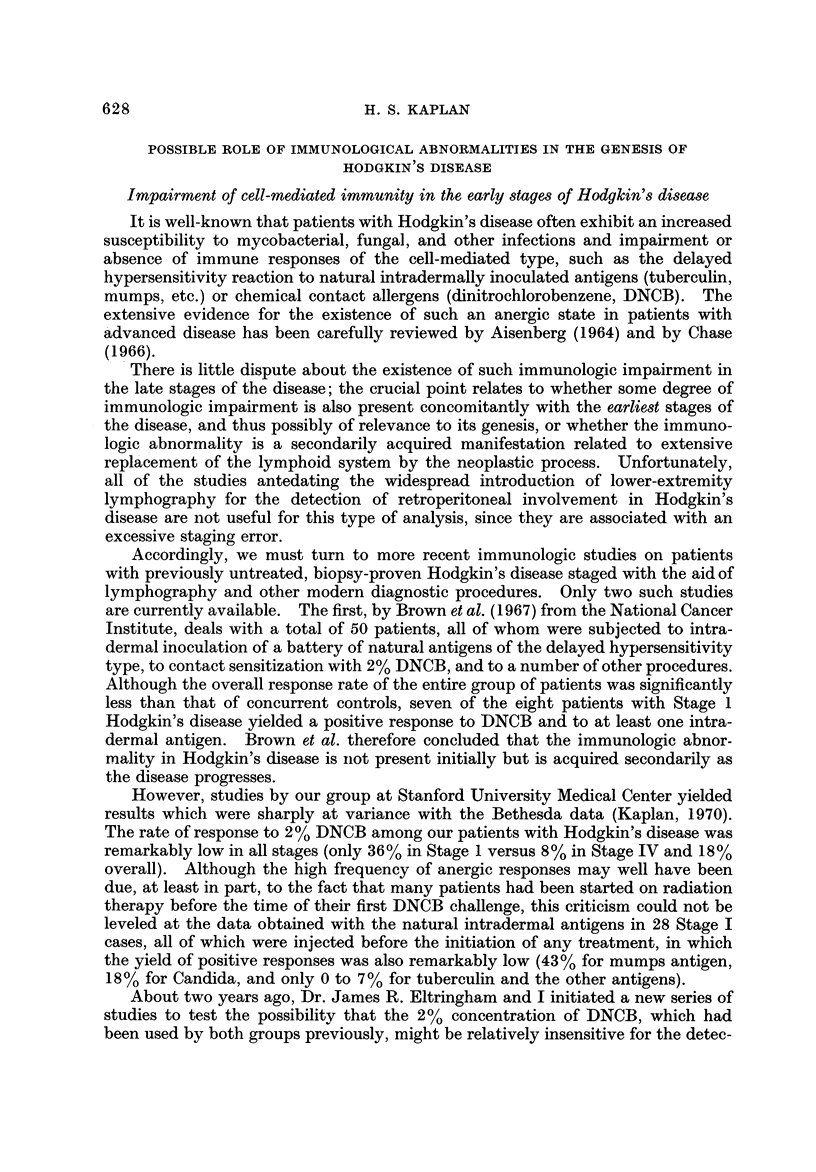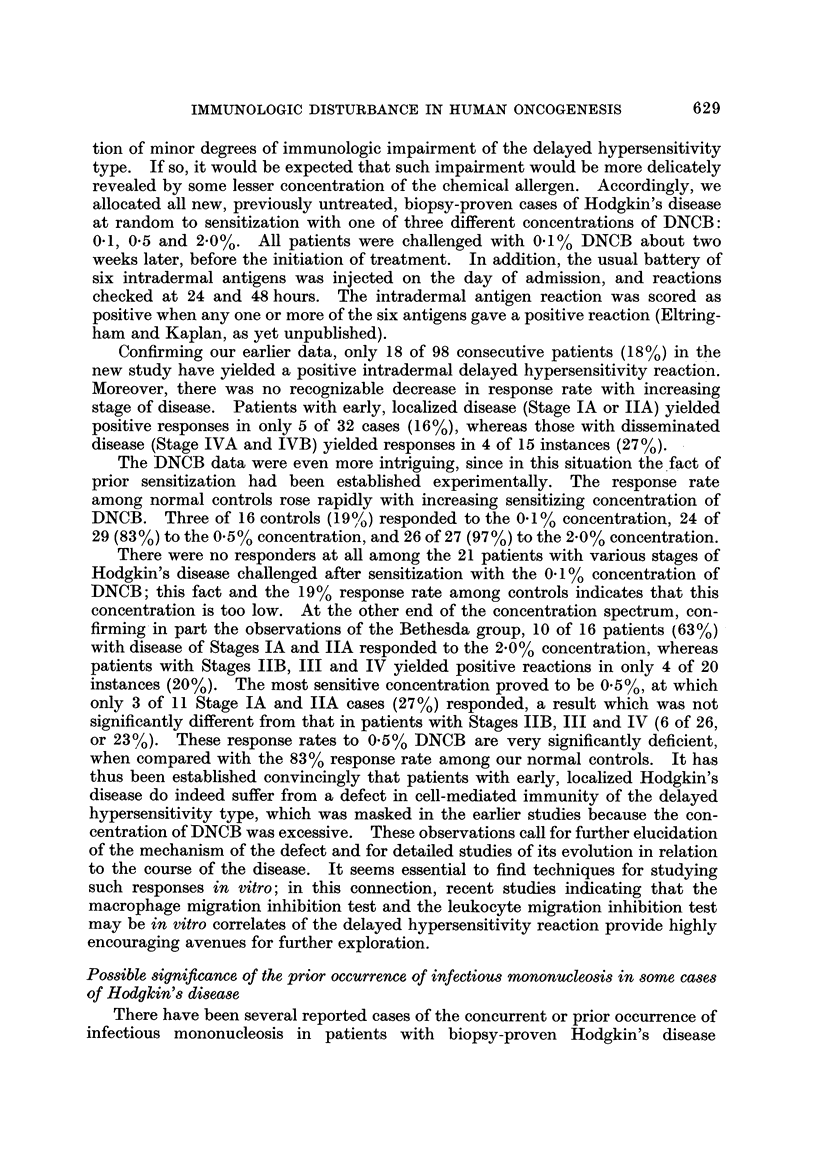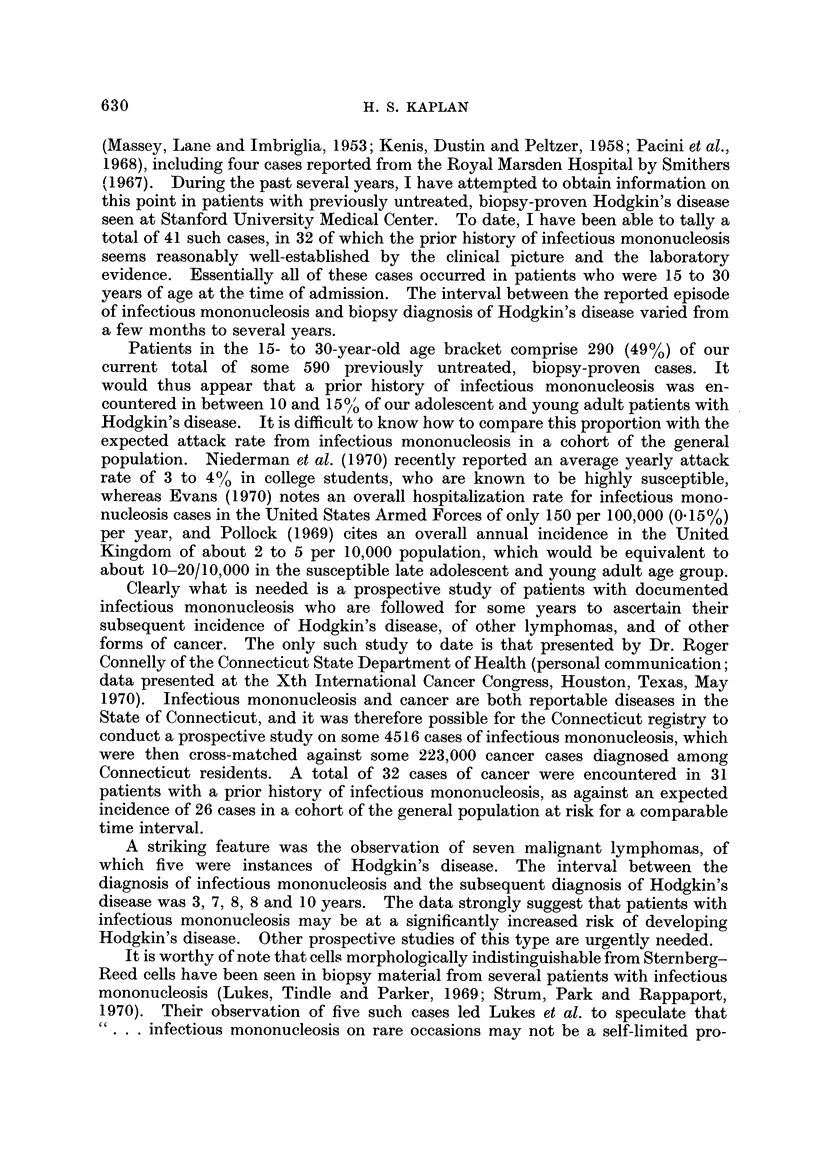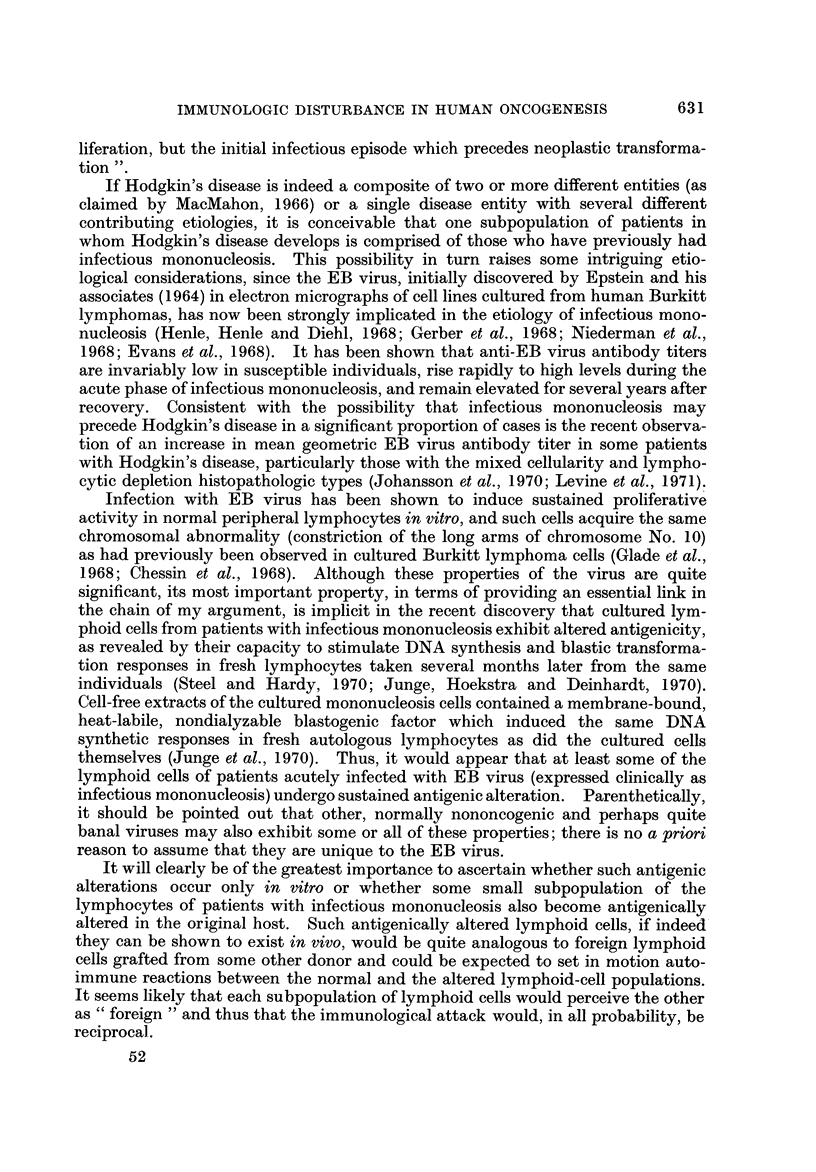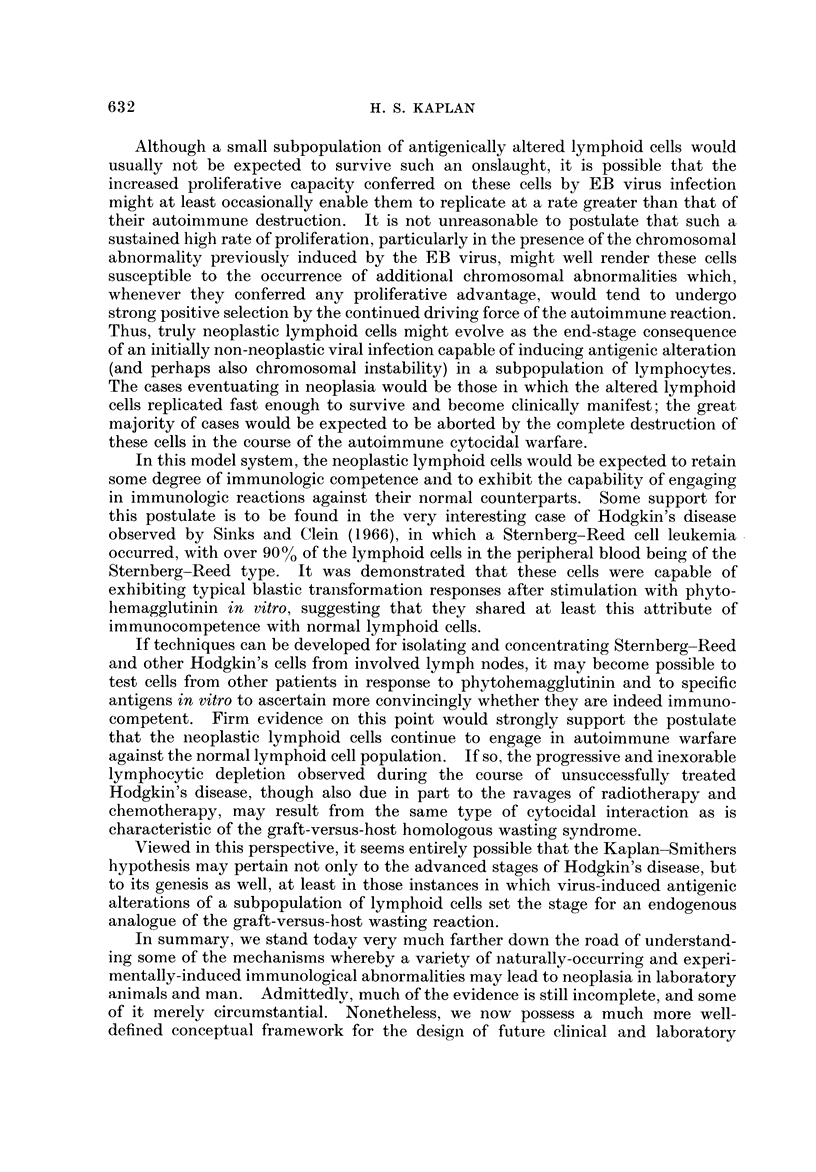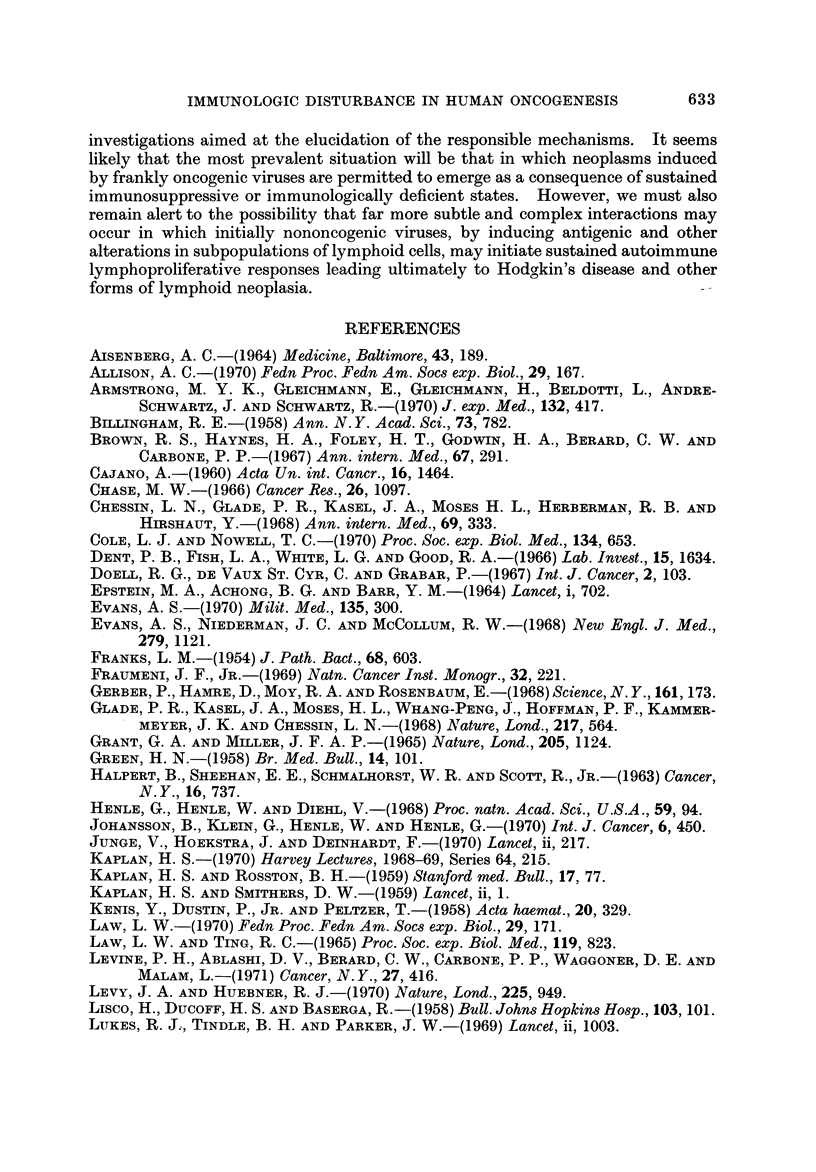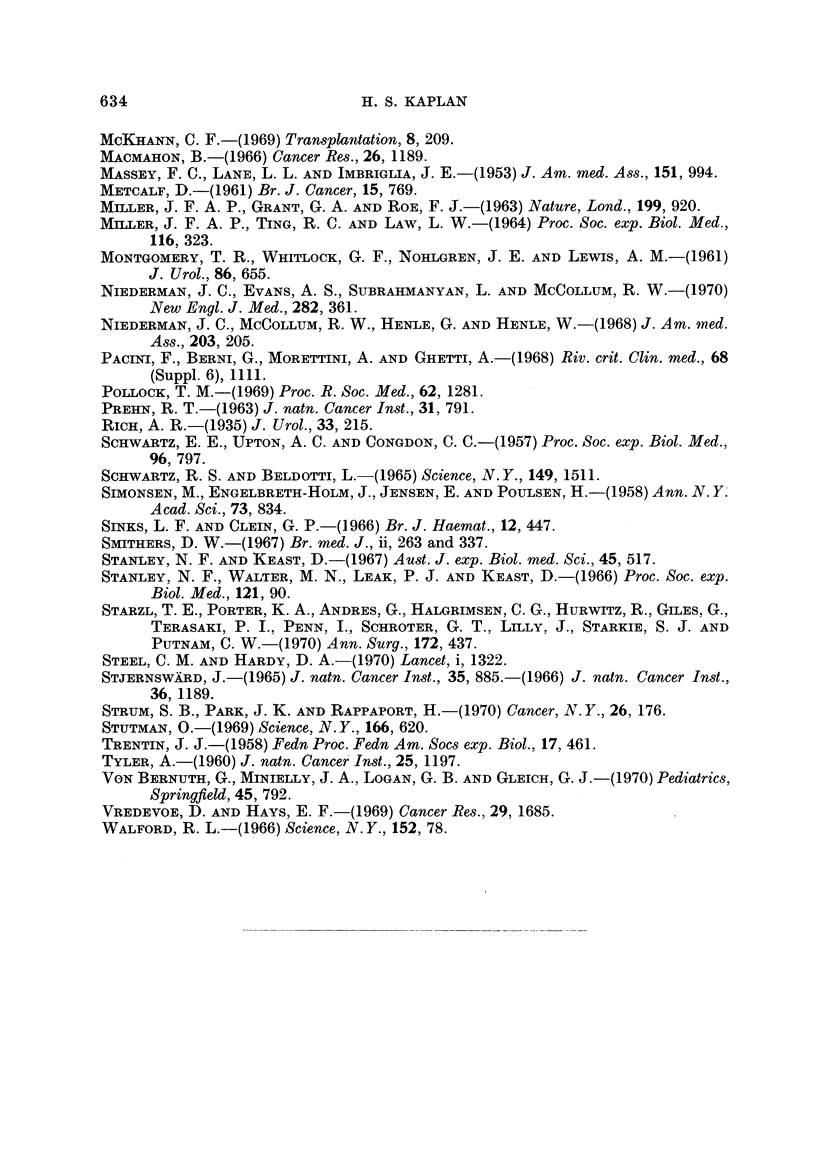# Role of Immunologic Disturbance in Human Oncogenesis: Some Facts and Fancies*

**DOI:** 10.1038/bjc.1971.78

**Published:** 1971-12

**Authors:** Henry S. Kaplan

## Abstract

A brief review is presented of the evidence linking the development of certain types of neoplasms, and of the malignant lymphomas in particular, to chronic immunosuppression in animals and man and to the naturally occurring human immunologic deficiency states. The discussion then focuses on Hodgkin's disease and considers recent evidence concerning the relation between the clinical stage of the disease and its associated defect in cell-mediated immunity. Finally, the prior occurrence of infectious mononucleosis in some cases of Hodgkin's disease is considered in the context of the hypothesis that the neoplastic cells of Hodgkin's disease may evolve from a chronic immunologic reaction, analogous to that of graft-versus-host, stemming from the induction of antigenic alteration in a subpopulation of lymphocytes by certain types of non-neoplastic viral infections.


### ROLE OF IMMUNOLOGIC DISTURBANCE IN HUMAN ONCOGENESIS: SOME FACTS AND FANCIES*

HENRY S. KAPLAN

*From the Department of Radiology, Stanford University School of Medicine, Stanford, California, U.S.A.*

Received for publication July 28, 1971

**SUMMARY.—A brief review is presented of the evidence linking the development of certain types of neoplasms, and of the malignant lymphomas in particular, to chronic immunosuppression in animals and man and to the naturally occurring human immunologic deficiency states. The discussion then focuses on Hodgkin's disease and considers recent evidence concerning the relation between the clinical stage of the disease and its associated defect in cell-mediated immunity. Finally, the prior occurrence of infectious mononucleosis in some cases of Hodgkin's disease is considered in the context of the hypothesis that the neoplastic cells of Hodgkin's disease may evolve from a chronic immunologic reaction, analogous to that of graft-versus-host, stemming from the induction of antigenic alteration in a subpopulation of lymphocytes by certain types of non-neoplastic viral infections.**

IMMUNOLOGIC theories of carcinogenesis are not new. As early as 1954, H. N. Green put forward such a theory, which invoked the loss of specific cellular antigen, secondary to binding of tissue proteins by carcinogens, as the essential cause of neoplasia (Green, 1958). Cajano (1960) proposed a concept of carcinogenesis which stressed the denaturation or unmasking of autoantigens, which thereafter behaved as foreign antigens, provoking a " chain reaction ".

Many of our present-day concepts of molecular biology, of the regulation of protein synthesis, and of the cellular and molecular mechanisms underlying immune responses were at that time in a relatively undeveloped state. It would have been surprising indeed if these early theories had remained valid in the light of modern knowledge. Nonetheless, they contained the germ of an important idea, namely, that immunologic mechanisms might contribute significantly to the genesis of neoplastic processes.

Two other early theories emphasized the role of graft-versus-host reactions of the type which, under other conditions, may lead to " homologous wasting disease", as a sustained stimulus for the proliferative activity of cancerous cells. One such theory, proposed by Tyler (1960), was a general one, relating to all types of cancers. It postulated the loss or inactivation of a gene or genes at one or more of the histocompatibility loci as the initiating event in carcinogenesis. Such gene loss or inactivation was believed to be the consequence of the action of

* The W. I. Hubert Lecture delivered April 1, 1971, at the annual meeting of the British Association for Cancer Research, Bristol, England. Clinical investigations from the author's department cited herein were supported with the aid of grant CA 05838 from the National Cancer Institute, National Institutes of Health, U.S. Public Health Service.

chemical, physical, or viral agents. The antigenically depleted tumor cells were considered capable of producing antibody against other cells of the " host ", and thus eliciting all of the essential pathological features of neoplasia. Tyler states: " On the basis of the assumption that tumor cells lack one or more ' host ' antigens, whereby the latter have become foreign to it, these cells are chronically exposed to ' foreign ' antigen. They would then be expected to proliferate indefinitely."

In short, the driving force for tumor cell replication was thought to reside in their sustained exposure to normal tissue antigens which, because of their own antigenic depletion, they now perceived as " foreign ".

A major difficulty with this theory is that it required that *all* types of potential tumor cells, including those of epithelial and non-lymphoid mesenchymal origin, be capable of activation to an immunoresponsive state under the special circumstances described. Although Tyler argued that non-lymphoid cells might undergo dedifferentiation and thus regain immunologic potentialities ordinarily reserved for lymphoid cells, no substantiation whatever for this aspect of his theory has emerged in the intervening decade.

A more specialized and limited theory was that proposed by Professor Sir David Smithers and myself (Kaplan and Smithers, 1959). We were impressed with the similarity between advanced forms of Hodgkin's disease in man and homologous graft-versus-host disease in animals with respect to such features as wasting, anaemia, and lymphocytic depletion. We postulated that tumor cells stemming from *lymphatic* tissue might well retain the capacity for immunologic responses and that under certain conditions such lymphoid tumor cells might become antigenically differentiated from the normal lymphoid cells of the individual, perhaps by " antigenic deletion ". We argued that: " They would then acquire the ability to react against and destroy the patient's normal lymphoid and other haematopoietic cells. The possibility of a two-way interaction cannot be excluded but seems less probable."

Such clinically observed sequelae as progressive lymphocytic depletion, hemolytic anaemia, wasting, and increased susceptibility to mycobacterial and fungal infection were regarded as entirely consonant with the concept that Hodgkin's disease may be an endogenous counterpart in man of graft-versus-host homologous disease in laboratory animals. It was clearly recognized that there were many gaps in our knowledge at that time which would have to be filled before this theory could be subjected to rigorous critical analysis.

Slightly more than a decade has now elapsed since these concepts were proposed. It seemed of interest to examine what we have learned concerning the role of immunologic phenomena in carcinogenesis generally and then to focus more specifically on Hodgkin's disease in an effort to ascertain whether any part of the Kaplan–Smithers hypothesis has withstood the test of time. The mere fact that we are gathered here for a symposium on " Immunosuppression and Carcinogenesis " is, in itself, a sufficient indication of the timeliness of such an analysis.

### IMPAIRMENT OF IMMUNOLOGIC SURVEILLANCE MECHANISMS PERMITTING OUTGROWTH OF TRANSFORMED CLONES

There is now ample evidence that clones of tumor cells attain microscopically detectable dimensions in certain tissues with a frequency which far exceeds the incidence of clinically apparent malignant neoplasms in those tissues. Perhaps the most outstanding example is the prostate gland. The work of Rich (1935), Franks (1954), and others (Halpert *et al.*, 1963) has revealed the presence of microcarcinomas in 15 to 30% or more of prostate glands examined at autopsy in males over 50 years of age dying of various causes. In contrast, the incidence of overt prostatic carcinoma in the maximally susceptible age range, 65 to 75 years, is only about 200 per 100,000 per year (0·2%). In a long-term follow-up study of 35 patients in whom microscopic evidence of prostatic cancer was discovered by chance after prostatic resection for benign obstructive disease, Montgomery *et al.* (1961) reported that none developed clinically evident prostatic cancer or died of prostatic cancer during the ensuing 8 years or more.

The very plausible suggestion has been put forward that immunologic surveillance mechanisms may be partly responsible for the prolonged growth restraint of many of these microscopic tumors, although it is recognized that the endocrine environment is almost certainly a major factor. On this view, it would be expected that the experimental induction of immunologic insufficiency in laboratory animals and the natural occurrence or iatrogenic induction of immunologic deficiency states in man would be followed by the development of an increased incidence of neoplasms. This prediction has been confirmed in a variety of experimental and clinical studies during the past decade. The following is a brief summary of the major observations to date.

*Enhanced susceptibility to neoplasms in neonatally thymectomized rodents*

The central role of the thymus gland in the development and maintenance of immunologic competence has been firmly established. Mice of several strains, when subjected to thymectomy in the neonatal period, develop a wasting syndrome morphologically indistinguishable from that induced by graft-versus-host reactions. Those that survive for any length of time exhibit severe immunologic impairment and a striking enhancement of susceptibility to the induction of neoplasms by polyoma and other oncogenic viruses (Miller, Ting and Law, 1964; Law and Ting, 1965). It has also been reported that they may exhibit a shorter latent period for tumor induction by the carcinogenic hydrocarbons, though the differences observed are not striking (Miller, Grant and Roe, 1963; Grant and Miller, 1965). Restoration of immunocompetence by the implantation of isologous thymic grafts simultaneously restores resistance to tumor induction by the oncogenic viruses (Law and Ting, 1965). It is thus clear that the development of the immune surveillance mechanism may be prevented by neonatal thymectomy and that the emergence of virus- and perhaps also of carcinogen-induced tumors is favored under these conditions.

*Immunosuppression by steroids, antimetabolites and antilymphocyte globulins (ALG)*

The yield of tumors induced in mice by polyoma and other oncogenic viruses can be sharply augmented by immunosuppressive treatment (Law, 1970; Allison, 1970). Such diverse agents as the adrenocortical steroids, the immunosuppressive antimetabolites, such as 6-mercaptopurine or azathioprine, and antilymphocyte globulins (Vredevoe and Hays, 1969; Law, 1970) have been effective. It has also been reported that the yield of spontaneous neoplasms may be enhanced in response to such treatment.

With the possible exception of 6-mercaptopurine (Doell *et al.*, 1967), there is no reason to believe that these immunosuppressive agents are carcinogenic in themselves; instead, we seem to be dealing with a situation in which microtumors induced by DNA tumor viruses, and perhaps by other agents, are permitted to grow to macroscopic dimensions by inhibition of the restraining influence of the immune surveillance system. In keeping with this interpretation, it has been shown that mice rendered susceptible to polyoma-induced neoplasia by immunosuppressive treatment can be restored to the normally resistant state by the injection of lymphoid cells from sensitized, immune donors, but not by lymphoid cells from normal donors (Allison, 1970).

Certain carcinogenic agents, notably ionizing radiations and the hydrocarbons, are themselves capable of producing significant injury and impairment of the immune system (Prehn, 1963; Stjernswärd, 1965, 1966; Doell *et al.*, 1967). Thus, such agents may have a *dual* action; in addition to inducing neoplastic transformation in clonogenic cells, either directly by mutation or indirectly through the activation of latent viruses, they may also create immunologic conditions favorable to the survival and sustained proliferation of these newly established, altered clones. However, two points are puzzling in this context. Firstly, the effect of the hydrocarbons seems to be limited to a transient depression of *humoral* antibody production, whereas one might have expected that the relevant effect would have been exerted on cell-mediated immunity. Secondly, there is the observation by Lisco *et al.* (1958), since confirmed by others, that sublethal whole-body X-ray exposures, which are undoubtedly immunosuppressive for both the humoral and cellular systems, fail to potentiate tumor induction by the hydrocarbons.

Limited data indicate that certain of the closely related *non*-carcinogenic hydrocarbons lack immunosuppressive activity (Stjernswärd, 1966). Moreover, it has been reported that a strain of mice known to be refractory to the carcinogenic action of hydrocarbons were also resistant to their immune suppressant effects (Stutman, 1969). However, in my view, careful further studies are needed to ascertain whether it is indeed a valid generalization, at least for certain classes of carcinogenic compounds, that the active members of the class are all immunosuppressive and that the inert ones are not. Rigorous evidence on this point will be needed before we can confidently conclude that some carcinogens owe their tumor-inducing activity in part to their capacity to inhibit immune processes.

### *Neoplasia in rodents with chronic graft-versus-host homologous disease*

It has long been known that graft-versus-host reactions induce an intense lymphoproliferative response of the sensitized graft cells (Simonsen *et al.*, 1958), essentially identical to the lymphoblastic transformation exhibited by lymphocytes *in vitro* after stimulation with specific antigens or nonspecific mitogens, such as phytohemagglutinin. The proliferative response is followed by a cytocidal attack upon the normal cells of the host, which is particularly manifest in the lymphoid tissues, leading to their progressive and profound depletion (Schwartz, Upton and Congdon, 1957; Billingham, 1958; Trentin, 1958; Kaplan and Rosston, 1959).

Although such reactions are usually acutely fatal in infant mice, some animals survive and slowly recover, though they may give evidence of a continuing smoldering reaction; adult animals tend to exhibit a more chronic, nonlethal form of the disease. Studying the long-term survivors among a population of $F_1$ hybrid mice in which graft-versus-host disease had been induced by the inoculation of parental strain spleen cells, Schwartz and Beldotti (1965) noted the early onset of a remarkably high incidence of malignant lymphomas, primarily of the type designated as the " reticulum cell neoplasm, type B ". An increased incidence of these tumors was also noted by Walford (1966) in mice injected with cells differing at weak histocompatibility loci. Schwartz and Beldotti were initially inclined to believe that these tumors might derive from the donor cells, in which case they could be interpreted as the neoplastic end result of a sustained immunoproliferative and lymphoproliferative stimulus. However, later studies clearly established that the tumors in their mice were all of host genotype (Armstrong *et al.*, 1970). Since the $F_1$ hybrid host cells in the system which they used were inherently incapable of reacting immunologically against the injected parental cells, their original hypothesis was clearly untenable. Despite the fact that their electron micrographic search for virus particles in these tumors was consistently unrewarding, the emergence of a latent oncogenic virus in these chronically immunosuppressed animals seems the most plausible alternative mechanism.

Some direct support for this alternative hypothesis has recently been forthcoming from another source. Stanley *et al.* (1966) reported the development of a transplantable malignant lymphoma, designated as 2731/L, in Prince Henry (PH) mice after inoculation with spleen cells from a 272-day-old PH mouse with chronic reovirus Type 3 infection. The fact that mice inoculated with reovirus Type 3 develop a " runting syndrome " essentially similar to other forms of homologous graft-versus-host disease suggested that this neoplasm may have developed on the same basis as those observed by Schwartz and Beldotti. Stanley and Keast (1967) demonstrated the persistent presence of reovirus antigen in the 2731/L tumor and were inclined to believe that reovirus had played a central role in its etiology. Recently, however, Levy and Huebner (1970) succeeded in isolating a murine leukemia virus with immunological characteristics indistinguishable from those of the Gross-AKR virus from the 2731/L tumor. They suggested that the tumor was probably induced by this Gross-type murine leukemia virus, and that the emergence of this virus in the spleen cells of the original reovirus-infected mice may well have been accelerated by the immunologic impairment associated with the runting syndrome from which they suffered. Clearly, careful further studies of this system are indicated, since the model of a nononcogenic virus inducing autoimmune disease, which in turn is followed by the emergence and oncogenic activity of a second, latent virus, has intriguing implications for man.

That the lymphoid tumors developing in mice surviving long-sustained homologous graft-versus-host reactions may, however, under certain conditions be due to the type of sustained immunoproliferative stimulus postulated by Smithers and myself, and later by Schwartz and Beldotti, is suggested by a recent report of Cole and Nowell (1970). Instead of using parental spleen cells and injecting them into nonirradiated $F_1$ hybrid mice, these investigators gave a single sublethal whole-body X-ray dose of 500 R to (C57L $\times$ A/He) $F_1$ hybrid mice, followed by the injection of either parental (A) lymph node cells (fresh or preincubated 2 hours at 37° C. *in vitro*) or of parental bone marrow, liver, or spleen cells. Although fresh lymph node cells yielded no tumors, 14 of 26 mice (54%) injected with preincubated lymph node cells developed tumors, an incidence significantly greater than that in their irradiated controls (17/132, or 13%). The tumors were described as " lymphosarcomas composed of sheets of medium and large lymphocytes, usually extrathymic in origin and involving the spleen, various lymph nodes, kidney, and liver. The most common variant consisted of more undifferentiated and pleomorphic cells shading into a reticulum cell pattern. Tumors resembling follicular lymphoma, Hodgkin's disease, and plasmacytoma were observed once or twice each ". Of particular interest was the high incidence of tumors developing in irradiated mice injected with parental bone marrow, liver, or spleen cells (13/17, or 76%; 8/10 or 80%; and 6/8 or 75%, respectively).

Several of the tumors developing under these conditions were clearly identified by their capacity to " take " and grow in adult A-strain hosts as being of donor genotype. It would appear, therefore, that the sustained immunoproliferative stimulation to which the donor cells were subjected must have played a role in the induction of these lymphoid tumors. This concept also derives support from the experiments of Metcalf (1961), who observed an increased incidence of reticular tumors in mice subjected to prolonged antigenic stimulation with *Salmonella* flagellar antigen or with bovine serum albumin. Curiously, Cole and Nowell report that their tumors failed to take in $F_1$ hybrid recipients; although they offer no explanation for this observation, it would suggest, if confirmed, that their lymphoid tumor cells may have retained their immunocompetence and thus were capable of reacting against the C57L histocompatibility antigens of the $F_1$ hybrid animals, presumably undergoing suicidal destruction in the process.

TABLE I.—*Neoplasia in Congenital Immunologic Deficiency Disorders (Fraumeni*, 1969)

| Disorder | Known or probable No. of cases at risk | No. of cases lymphoma | No. of cases leukemia |
|---|---|---|---|
| 1. Ataxia-telangiectasia | 200 (?) . | 13 (3 sibs) | 3 |
| 2. Wiskott–Aldrich syndrome | 60+ (1967) . | 6 . | 1 |
| 3. Agammaglobulinemia, | 24 (1963) . | 1 . | 1 |
| hypogammaglobulinemia, thymic aplasia | ? . | 2 . | 1 |
| 4. Chediak–Higashi syndrome | 36 (1967) . | 4 (?) . | — |

*Neoplasia in human congenital immunologic deficiency disorders*

A widening spectrum of rare congenital immunologic deficiency states has now been described. Some of these experiments of nature have been likened to the syndrome induced by neonatal thymectomy in mice, others to that resulting from bursectomy in chickens, and some are not yet fully characterized. Isolated case reports first called attention to the occurrence in some of these unfortunate patients of malignant neoplasms. In the course of time, careful collations of the literature have yielded enough data to make it clear that patients with congenital immunologic deficiency disorders are indeed at a sharply increased risk of developing such neoplasms. From the recent careful analysis by Fraumeni (1969), it is apparent that the type of neoplasm most often encountered in this situation is a malignant lymphoma, rather than a leukemia or epithelial neoplasm, and there is some evidence to suggest that diffuse histiocytic lymphomas are the principal variant within the lymphoma category (Table I). There is still uncertainty, however, with respect to the peculiar lesion observed in patients with the Chediak–Higashi anomaly; it is clearly some form of lymphoproliferative condition, but whether it can appropriately be designated a true neoplasm remains uncertain (Dent *et al.*, 1966).

A remarkable case has recently been reported by von Bernuth *et al.* (1970) of Hodgkin's disease encountered at autopsy in a 5-month-old infant with thymic aplasia and agammaglobulinemia. At Stanford University Medical Center, we have recently observed two 10-year old male patients with hypogammaglobulinemia (one deficient in IgA, the other in both IgA and IgG, with an abnormal IgM). These two boys exhibited a chronic reactive lymphadenopathy for several years, which finally progressed to frankly neoplastic proliferation and dissemination. Although the initial biopsies were interpreted as Hodgkin's disease in both instances, due to the presence of large binucleate cells morphologically indistinguishable from Sternberg–Reed cells, careful review of additional biopsy material in one case and of autopsy material in the other resulted in reclassification of these cases, one as a diffuse histiocytic lymphoma, the other as a " lymphoma, unclassified, most closely resembling lymphocyte predominant Hodgkin's disease ".

TABLE II.—*Primary Malignant Neoplasms in Immunosuppressed Renal Transplant Recipients* (*McKhann*, 1969)

Estimated total number of cases recorded in renal transplant registry = 2000. Number of primary neoplasms reported to date = 14

Types of primary neoplasms reported:

| | | | |
|---|---|---|---|
| RCSA | = 5 | Anaplastic Ca | = 2 |
| LSA | = 1 | Squamous cell Ca | = 2 |
| " Lymphoma " | = 1 | Dysgerminoma | = 1 |
| Plasmacytoma | = 1 | | |
| " Leukemia " | = 1 | | |
| | 9 | | 5 |

Estimates of incidence (per 100,000):

| | All types all ages | All types age < 40 | RCSA |
|---|---|---|---|
| General population | 130 | 8·2 | 0·9 |
| Renal transplant cases | 650 | 500 | 400 |

*Malignant neoplasms in immunosuppressed renal transplant recipients*

The rejection of renal homografts transplanted to patients with severe renal insufficiency has been prevented with increasing success by the chronic administration of azathioprine, supplemented intermittently by steroids, and the prolongation of useful life thus achieved has been gratifying indeed. However, it is now becoming clear that one of the prices that will have to be paid for this medical advance is an increased susceptibility to the development of malignant neoplasms in the chronically antigen-exposed, immunosuppressed transplant recipients. After several isolated case reports had appeared in the literature, McKhann (1969) distributed a questionnaire to many departments of surgery known to have active renal transplantation programs. He added the cases reported by those responding to the questionnaire to those in the published literature to obtain a total of 14 cases among an estimated total of 2000 transplant recipients. The types of tumor recorded in this initial survey and the crude estimates of incidence as compared with that in the general population for all malignancies and for reticulum cell sarcoma (diffuse histiocytic lymphoma) are summarized in Table II. This tabulation excludes other cases of cancer which were transplanted inadvertently from donors, as well as cases in which the transplant recipients were known to have had a pre-existing cancer. A particularly striking feature of McKhann's survey was the occurrence of a very high incidence of neoplasms in individuals under 40 years of age.

More recently, Starzl *et al.* (1970), in a comprehensive analysis of long-term survival after renal transplantation in man, noted the occurrence of 10 instances of primary malignant tumors (7 epithelial, 3 mesenchymal) among the 189 patients in their series. After allowance for early deaths, the net number of patients at risk was estimated at 140, yielding an adjusted tumor incidence of 7%. In the age range represented by their series of patients (5 to 49 years, with an average of 32 years), a cohort from the general population would have been expected to exhibit a total yearly cancer incidence of about 0·06%, suggesting that the risk in their chronically immunosuppressed renal transplant patients had been enhanced about 100-fold (Table III). Starzl and his colleagues also collated case reports from the literature and through personal inquiry to obtain a total of 27

TABLE III.—*Primary Malignant Neoplasms in Immunosuppressed Renal Transplant Recipients* (*Starzl* et al., 1970)

| | Crude No. at risk | Net No. at risk | Neoplasms | | | Incidence, % | |
|---|---|---|---|---|---|---|---|
| | | | Epith. | Mesench. | Total | Crude | Net |
| 1. Starzl *et al.* (age range 5–49, average 32 years) | 189 | 140 | 7 | 3 | 10 | 5·3 | 7·1 |
| | | | Expected in cohort of general population | | | | 0·06 |
| 2. Other published cases | — | — | 14 | 13 | 27 | — | — |
| | | Totals | 21 | 16 | 37 | | |

Histiocytic lymphoma (" RCSA ") in 12 of 16 mesenchymal tumors; usually early in onset (range 5–67, average 21 months) and with very unfavorable prognosis (11/12 fatal).

additional cases from other departments of surgery. Epithelial neoplasms account for 21 of the combined total of 37 malignancies, as might be expected from their far greater prevalence. However, 12 of the 16 mesenchymal neoplasms were reticulum cell sarcomas (diffuse histiocytic lymphomas). Most of these occurred in young patients, and tended to develop relatively early in the post-operative period; the observed latent periods ranged from 5 to 67 months, with an average of only 21 months. Whereas the epithelial neoplasms exhibited a reasonably favorable prognosis in response to conventional treatment, the reticulum cell sarcomas had an extremely poor prognosis, 11 of the 12 recorded cases terminating fatally.

It should be noted that there is no proof that these neoplasms have developed solely as a consequence of chronic immunosuppression *per se.* These patients were also chronically exposed to foreign antigen from the donor kidney, and it is thus entirely possible that the lymphomas, like those in Metcalf's mice, arose in response to sustained immunologic stimulation. Moreover, if immune surveillance is indeed important in restraining the evolution of prostatic microtumors, it is curious that there have been no prostatic cancers reported to date; however, the relatively young age of most of the renal transplant recipients may explain their absence.

### POSSIBLE ROLE OF IMMUNOLOGICAL ABNORMALITIES IN THE GENESIS OF HODGKIN'S DISEASE

### *Impairment of cell-mediated immunity in the early stages of Hodgkin's disease*

It is well-known that patients with Hodgkin's disease often exhibit an increased susceptibility to mycobacterial, fungal, and other infections and impairment or absence of immune responses of the cell-mediated type, such as the delayed hypersensitivity reaction to natural intradermally inoculated antigens (tuberculin, mumps, etc.) or chemical contact allergens (dinitrochlorobenzene, DNCB). The extensive evidence for the existence of such an anergic state in patients with advanced disease has been carefully reviewed by Aisenberg (1964) and by Chase (1966).

There is little dispute about the existence of such immunologic impairment in the late stages of the disease; the crucial point relates to whether some degree of immunologic impairment is also present concomitantly with the *earliest* stages of the disease, and thus possibly of relevance to its genesis, or whether the immunologic abnormality is a secondarily acquired manifestation related to extensive replacement of the lymphoid system by the neoplastic process. Unfortunately, all of the studies antedating the widespread introduction of lower-extremity lymphography for the detection of retroperitoneal involvement in Hodgkin's disease are not useful for this type of analysis, since they are associated with an excessive staging error.

Accordingly, we must turn to more recent immunologic studies on patients with previously untreated, biopsy-proven Hodgkin's disease staged with the aid of lymphography and other modern diagnostic procedures. Only two such studies are currently available. The first, by Brown *et al.* (1967) from the National Cancer Institute, deals with a total of 50 patients, all of whom were subjected to intradermal inoculation of a battery of natural antigens of the delayed hypersensitivity type, to contact sensitization with 2% DNCB, and to a number of other procedures. Although the overall response rate of the entire group of patients was significantly less than that of concurrent controls, seven of the eight patients with Stage 1 Hodgkin's disease yielded a positive response to DNCB and to at least one intradermal antigen. Brown *et al.* therefore concluded that the immunologic abnormality in Hodgkin's disease is not present initially but is acquired secondarily as the disease progresses.

However, studies by our group at Stanford University Medical Center yielded results which were sharply at variance with the Bethesda data (Kaplan, 1970). The rate of response to 2% DNCB among our patients with Hodgkin's disease was remarkably low in all stages (only 36% in Stage 1 versus 8% in Stage IV and 18% overall). Although the high frequency of anergic responses may well have been due, at least in part, to the fact that many patients had been started on radiation therapy before the time of their first DNCB challenge, this criticism could not be leveled at the data obtained with the natural intradermal antigens in 28 Stage I cases, all of which were injected before the initiation of any treatment, in which the yield of positive responses was also remarkably low (43% for mumps antigen, 18% for Candida, and only 0 to 7% for tuberculin and the other antigens).

About two years ago, Dr. James R. Eltringham and I initiated a new series of studies to test the possibility that the 2% concentration of DNCB, which had been used by both groups previously, might be relatively insensitive for the detection of minor degrees of immunologic impairment of the delayed hypersensitivity type. If so, it would be expected that such impairment would be more delicately revealed by some lesser concentration of the chemical allergen. Accordingly, we allocated all new, previously untreated, biopsy-proven cases of Hodgkin's disease at random to sensitization with one of three different concentrations of DNCB: 0·1, 0·5 and 2·0%. All patients were challenged with 0·1% DNCB about two weeks later, before the initiation of treatment. In addition, the usual battery of six intradermal antigens was injected on the day of admission, and reactions checked at 24 and 48 hours. The intradermal antigen reaction was scored as positive when any one or more of the six antigens gave a positive reaction (Eltringham and Kaplan, as yet unpublished).

Confirming our earlier data, only 18 of 98 consecutive patients (18%) in the new study have yielded a positive intradermal delayed hypersensitivity reaction. Moreover, there was no recognizable decrease in response rate with increasing stage of disease. Patients with early, localized disease (Stage IA or IIA) yielded positive responses in only 5 of 32 cases (16%), whereas those with disseminated disease (Stage IVA and IVB) yielded responses in 4 of 15 instances (27%).

The DNCB data were even more intriguing, since in this situation the fact of prior sensitization had been established experimentally. The response rate among normal controls rose rapidly with increasing sensitizing concentration of DNCB. Three of 16 controls (19%) responded to the 0·1% concentration, 24 of 29 (83%) to the 0·5% concentration, and 26 of 27 (97%) to the 2·0% concentration.

There were no responders at all among the 21 patients with various stages of Hodgkin's disease challenged after sensitization with the 0·1% concentration of DNCB; this fact and the 19% response rate among controls indicates that this concentration is too low. At the other end of the concentration spectrum, confirming in part the observations of the Bethesda group, 10 of 16 patients (63%) with disease of Stages IA and IIA responded to the 2·0% concentration, whereas patients with Stages IIB, III and IV yielded positive reactions in only 4 of 20 instances (20%). The most sensitive concentration proved to be 0·5%, at which only 3 of 11 Stage IA and IIA cases (27%) responded, a result which was not significantly different from that in patients with Stages IIB, III and IV (6 of 26, or 23%). These response rates to 0·5% DNCB are very significantly deficient, when compared with the 83% response rate among our normal controls. It has thus been established convincingly that patients with early, localized Hodgkin's disease do indeed suffer from a defect in cell-mediated immunity of the delayed hypersensitivity type, which was masked in the earlier studies because the concentration of DNCB was excessive. These observations call for further elucidation of the mechanism of the defect and for detailed studies of its evolution in relation to the course of the disease. It seems essential to find techniques for studying such responses *in vitro*; in this connection, recent studies indicating that the macrophage migration inhibition test and the leukocyte migration inhibition test may be *in vitro* correlates of the delayed hypersensitivity reaction provide highly encouraging avenues for further exploration.

*Possible significance of the prior occurrence of infectious mononucleosis in some cases of Hodgkin's disease*

There have been several reported cases of the concurrent or prior occurrence of infectious mononucleosis in patients with biopsy-proven Hodgkin's disease

(Massey, Lane and Imbriglia, 1953; Kenis, Dustin and Peltzer, 1958; Pacini *et al.*, 1968), including four cases reported from the Royal Marsden Hospital by Smithers (1967). During the past several years, I have attempted to obtain information on this point in patients with previously untreated, biopsy-proven Hodgkin's disease seen at Stanford University Medical Center. To date, I have been able to tally a total of 41 such cases, in 32 of which the prior history of infectious mononucleosis seems reasonably well-established by the clinical picture and the laboratory evidence. Essentially all of these cases occurred in patients who were 15 to 30 years of age at the time of admission. The interval between the reported episode of infectious mononucleosis and biopsy diagnosis of Hodgkin's disease varied from a few months to several years.

Patients in the 15- to 30-year-old age bracket comprise 290 (49%) of our current total of some 590 previously untreated, biopsy-proven cases. It would thus appear that a prior history of infectious mononucleosis was encountered in between 10 and 15% of our adolescent and young adult patients with Hodgkin's disease. It is difficult to know how to compare this proportion with the expected attack rate from infectious mononucleosis in a cohort of the general population. Niederman *et al.* (1970) recently reported an average yearly attack rate of 3 to 4% in college students, who are known to be highly susceptible, whereas Evans (1970) notes an overall hospitalization rate for infectious mononucleosis cases in the United States Armed Forces of only 150 per 100,000 (0·15%) per year, and Pollock (1969) cites an overall annual incidence in the United Kingdom of about 2 to 5 per 10,000 population, which would be equivalent to about 10–20/10,000 in the susceptible late adolescent and young adult age group.

Clearly what is needed is a prospective study of patients with documented infectious mononucleosis who are followed for some years to ascertain their subsequent incidence of Hodgkin's disease, of other lymphomas, and of other forms of cancer. The only such study to date is that presented by Dr. Roger Connelly of the Connecticut State Department of Health (personal communication; data presented at the Xth International Cancer Congress, Houston, Texas, May 1970). Infectious mononucleosis and cancer are both reportable diseases in the State of Connecticut, and it was therefore possible for the Connecticut registry to conduct a prospective study on some 4516 cases of infectious mononucleosis, which were then cross-matched against some 223,000 cancer cases diagnosed among Connecticut residents. A total of 32 cases of cancer were encountered in 31 patients with a prior history of infectious mononucleosis, as against an expected incidence of 26 cases in a cohort of the general population at risk for a comparable time interval.

A striking feature was the observation of seven malignant lymphomas, of which five were instances of Hodgkin's disease. The interval between the diagnosis of infectious mononucleosis and the subsequent diagnosis of Hodgkin's disease was 3, 7, 8, 8 and 10 years. The data strongly suggest that patients with infectious mononucleosis may be at a significantly increased risk of developing Hodgkin's disease. Other prospective studies of this type are urgently needed.

It is worthy of note that cells morphologically indistinguishable from Sternberg–Reed cells have been seen in biopsy material from several patients with infectious mononucleosis (Lukes, Tindle and Parker, 1969; Strum, Park and Rappaport, 1970). Their observation of five such cases led Lukes *et al.* to speculate that " . . . infectious mononucleosis on rare occasions may not be a self-limited proliferation, but the initial infectious episode which precedes neoplastic transformation ".

If Hodgkin's disease is indeed a composite of two or more different entities (as claimed by MacMahon, 1966) or a single disease entity with several different contributing etiologies, it is conceivable that one subpopulation of patients in whom Hodgkin's disease develops is comprised of those who have previously had infectious mononucleosis. This possibility in turn raises some intriguing etiological considerations, since the EB virus, initially discovered by Epstein and his associates (1964) in electron micrographs of cell lines cultured from human Burkitt lymphomas, has now been strongly implicated in the etiology of infectious mononucleosis (Henle, Henle and Diehl, 1968; Gerber *et al.*, 1968; Niederman *et al.*, 1968; Evans *et al.*, 1968). It has been shown that anti-EB virus antibody titers are invariably low in susceptible individuals, rise rapidly to high levels during the acute phase of infectious mononucleosis, and remain elevated for several years after recovery. Consistent with the possibility that infectious mononucleosis may precede Hodgkin's disease in a significant proportion of cases is the recent observation of an increase in mean geometric EB virus antibody titer in some patients with Hodgkin's disease, particularly those with the mixed cellularity and lymphocytic depletion histopathologic types (Johansson *et al.*, 1970; Levine *et al.*, 1971).

Infection with EB virus has been shown to induce sustained proliferative activity in normal peripheral lymphocytes *in vitro*, and such cells acquire the same chromosomal abnormality (constriction of the long arms of chromosome No. 10) as had previously been observed in cultured Burkitt lymphoma cells (Glade *et al.*, 1968; Chessin *et al.*, 1968). Although these properties of the virus are quite significant, its most important property, in terms of providing an essential link in the chain of my argument, is implicit in the recent discovery that cultured lymphoid cells from patients with infectious mononucleosis exhibit altered antigenicity, as revealed by their capacity to stimulate DNA synthesis and blastic transformation responses in fresh lymphocytes taken several months later from the same individuals (Steel and Hardy, 1970; Junge, Hoekstra and Deinhardt, 1970). Cell-free extracts of the cultured mononucleosis cells contained a membrane-bound, heat-labile, nondialyzable blastogenic factor which induced the same DNA synthetic responses in fresh autologous lymphocytes as did the cultured cells themselves (Junge *et al.*, 1970). Thus, it would appear that at least some of the lymphoid cells of patients acutely infected with EB virus (expressed clinically as infectious mononucleosis) undergo sustained antigenic alteration. Parenthetically, it should be pointed out that other, normally nononcogenic and perhaps quite banal viruses may also exhibit some or all of these properties; there is no *a priori* reason to assume that they are unique to the EB virus.

It will clearly be of the greatest importance to ascertain whether such antigenic alterations occur only *in vitro* or whether some small subpopulation of the lymphocytes of patients with infectious mononucleosis also become antigenically altered in the original host. Such antigenically altered lymphoid cells, if indeed they can be shown to exist *in vivo*, would be quite analogous to foreign lymphoid cells grafted from some other donor and could be expected to set in motion autoimmune reactions between the normal and the altered lymphoid-cell populations. It seems likely that each subpopulation of lymphoid cells would perceive the other as " foreign " and thus that the immunological attack would, in all probability, be reciprocal.

Although a small subpopulation of antigenically altered lymphoid cells would usually not be expected to survive such an onslaught, it is possible that the increased proliferative capacity conferred on these cells by EB virus infection might at least occasionally enable them to replicate at a rate greater than that of their autoimmune destruction. It is not unreasonable to postulate that such a sustained high rate of proliferation, particularly in the presence of the chromosomal abnormality previously induced by the EB virus, might well render these cells susceptible to the occurrence of additional chromosomal abnormalities which, whenever they conferred any proliferative advantage, would tend to undergo strong positive selection by the continued driving force of the autoimmune reaction. Thus, truly neoplastic lymphoid cells might evolve as the end-stage consequence of an initially non-neoplastic viral infection capable of inducing antigenic alteration (and perhaps also chromosomal instability) in a subpopulation of lymphocytes. The cases eventuating in neoplasia would be those in which the altered lymphoid cells replicated fast enough to survive and become clinically manifest; the great majority of cases would be expected to be aborted by the complete destruction of these cells in the course of the autoimmune cytocidal warfare.

In this model system, the neoplastic lymphoid cells would be expected to retain some degree of immunologic competence and to exhibit the capability of engaging in immunologic reactions against their normal counterparts. Some support for this postulate is to be found in the very interesting case of Hodgkin's disease observed by Sinks and Clein (1966), in which a Sternberg–Reed cell leukemia occurred, with over 90% of the lymphoid cells in the peripheral blood being of the Sternberg–Reed type. It was demonstrated that these cells were capable of exhibiting typical blastic transformation responses after stimulation with phytohemagglutinin *in vitro*, suggesting that they shared at least this attribute of immunocompetence with normal lymphoid cells.

If techniques can be developed for isolating and concentrating Sternberg–Reed and other Hodgkin's cells from involved lymph nodes, it may become possible to test cells from other patients in response to phytohemagglutinin and to specific antigens *in vitro* to ascertain more convincingly whether they are indeed immunocompetent. Firm evidence on this point would strongly support the postulate that the neoplastic lymphoid cells continue to engage in autoimmune warfare against the normal lymphoid cell population. If so, the progressive and inexorable lymphocytic depletion observed during the course of unsuccessfully treated Hodgkin's disease, though also due in part to the ravages of radiotherapy and chemotherapy, may result from the same type of cytocidal interaction as is characteristic of the graft-versus-host homologous wasting syndrome.

Viewed in this perspective, it seems entirely possible that the Kaplan–Smithers hypothesis may pertain not only to the advanced stages of Hodgkin's disease, but to its genesis as well, at least in those instances in which virus-induced antigenic alterations of a subpopulation of lymphoid cells set the stage for an endogenous analogue of the graft-versus-host wasting reaction.

In summary, we stand today very much farther down the road of understanding some of the mechanisms whereby a variety of naturally-occurring and experimentally-induced immunological abnormalities may lead to neoplasia in laboratory animals and man. Admittedly, much of the evidence is still incomplete, and some of it merely circumstantial. Nonetheless, we now possess a much more well-defined conceptual framework for the design of future clinical and laboratory investigations aimed at the elucidation of the responsible mechanisms. It seems likely that the most prevalent situation will be that in which neoplasms induced by frankly oncogenic viruses are permitted to emerge as a consequence of sustained immunosuppressive or immunologically deficient states. However, we must also remain alert to the possibility that far more subtle and complex interactions may occur in which initially nononcogenic viruses, by inducing antigenic and other alterations in subpopulations of lymphoid cells, may initiate sustained autoimmune lymphoproliferative responses leading ultimately to Hodgkin's disease and other forms of lymphoid neoplasia.

### REFERENCES

AISENBERG, A. C.—(1964) *Medicine, Baltimore*, **43**, 189.
ALLISON, A. C.—(1970) *Fedn Proc. Fedn Am. Socs exp. Biol.*, **29**, 167.
ARMSTRONG, M. Y. K., GLEICHMANN, E., GLEICHMANN, H., BELDOTTI, L., ANDRE-SCHWARTZ, J. AND SCHWARTZ, R.—(1970) *J. exp. Med.*, **132**, 417.
BILLINGHAM, R. E.—(1958) *Ann. N.Y. Acad. Sci.*, **73**, 782.
BROWN, R. S., HAYNES, H. A., FOLEY, H. T., GODWIN, H. A., BERARD, C. W. AND CARBONE, P. P.—(1967) *Ann. intern. Med.*, **67**, 291.
CAJANO, A.—(1960) *Acta Un. int. Cancr.*, **16**, 1464.
CHASE, M. W.—(1966) *Cancer Res.*, **26**, 1097.
CHESSIN, L. N., GLADE, P. R., KASEL, J. A., MOSES H. L., HERBERMAN, R. B. AND HIRSHAUT, Y.—(1968) *Ann. intern. Med.*, **69**, 333.
COLE, L. J. AND NOWELL, T. C.—(1970) *Proc. Soc. exp. Biol. Med.*, **134**, 653.
DENT, P. B., FISH, L. A., WHITE, L. G. AND GOOD, R. A.—(1966) *Lab. Invest.*, **15**, 1634.
DOELL, R. G., DE VAUX ST. CYR, C. AND GRABAR, P.—(1967) *Int. J. Cancer*, **2**, 103.
EPSTEIN, M. A., ACHONG, B. G. AND BARR, Y. M.—(1964) *Lancet*, i, 702.
EVANS, A. S.—(1970) *Milit. Med.*, **135**, 300.
EVANS, A. S., NIEDERMAN, J. C. AND MCCOLLUM, R. W.—(1968) *New Engl. J. Med.*, **279**, 1121.
FRANKS, L. M.—(1954) *J. Path. Bact.*, **68**, 603.
FRAUMENI, J. F., JR.—(1969) *Natn. Cancer Inst. Monogr.*, **32**, 221.
GERBER, P., HAMRE, D., MOY, R. A. AND ROSENBAUM, E.—(1968) *Science, N.Y.*, **161**, 173.
GLADE, P. R., KASEL, J. A., MOSES, H. L., WHANG-PENG, J., HOFFMAN, P. F., KAMMERMEYER, J. K. AND CHESSIN, L. N.—(1968) *Nature, Lond.*, **217**, 564.
GRANT, G. A. AND MILLER, J. F. A. P.—(1965) *Nature, Lond.*, **205**, 1124.
GREEN, H. N.—(1958) *Br. Med. Bull.*, **14**, 101.
HALPERT, B., SHEEHAN, E. E., SCHMALHORST, W. R. AND SCOTT, R., JR.—(1963) *Cancer, N.Y.*, **16**, 737.
HENLE, G., HENLE, W. AND DIEHL, V.—(1968) *Proc. natn. Acad. Sci., U.S.A.*, **59**, 94.
JOHANSSON, B., KLEIN, G., HENLE, W. AND HENLE, G.—(1970) *Int. J. Cancer*, **6**, 450.
JUNGE, V., HOEKSTRA, J. AND DEINHARDT, F.—(1970) *Lancet*, ii, 217.
KAPLAN, H. S.—(1970) *Harvey Lectures*, 1968–69, Series 64, 215.
KAPLAN, H. S. AND ROSSTON, B. H.—(1959) *Stanford med. Bull.*, **17**, 77.
KAPLAN, H. S. AND SMITHERS, D. W.—(1959) *Lancet*, ii, 1.
KENIS, Y., DUSTIN, P., JR. AND PELTZER, T.—(1958) *Acta haemat.*, **20**, 329.
LAW, L. W.—(1970) *Fedn Proc. Fedn Am. Socs exp. Biol.*, **29**, 171.
LAW, L. W. AND TING, R. C.—(1965) *Proc. Soc. exp. Biol. Med.*, **119**, 823.
LEVINE, P. H., ABLASHI, D. V., BERARD, C. W., CARBONE, P. P., WAGGONER, D. E. AND MALAM, L.—(1971) *Cancer, N.Y.*, **27**, 416.
LEVY, J. A. AND HUEBNER, R. J.—(1970) *Nature, Lond.*, **225**, 949.
LISCO, H., DUCOFF, H. S. AND BASERGA, R.—(1958) *Bull. Johns Hopkins Hosp.*, **103**, 101.
LUKES, R. J., TINDLE, B. H. AND PARKER, J. W.—(1969) *Lancet*, ii, 1003.

McKhann, C. F.—(1969) *Transplantation*, **8**, 209.
MacMahon, B.—(1966) *Cancer Res.*, **26**, 1189.
Massey, F. C., Lane, L. L. and Imbriglia, J. E.—(1953) *J. Am. med. Ass.*, **151**, 994.
Metcalf, D.—(1961) *Br. J. Cancer*, **15**, 769.
Miller, J. F. A. P., Grant, G. A. and Roe, F. J.—(1963) *Nature, Lond.*, **199**, 920.
Miller, J. F. A. P., Ting, R. C. and Law, L. W.—(1964) *Proc. Soc. exp. Biol. Med.*, **116**, 323.
Montgomery, T. R., Whitlock, G. F., Nohlgren, J. E. and Lewis, A. M.—(1961) *J. Urol.*, **86**, 655.
Niederman, J. C., Evans, A. S., Subrahmanyan, L. and McCollum, R. W.—(1970) *New Engl. J. Med.*, **282**, 361.
Niederman, J. C., McCollum, R. W., Henle, G. and Henle, W.—(1968) *J. Am. med. Ass.*, **203**, 205.
Pacini, F., Berni, G., Morettini, A. and Ghetti, A.—(1968) *Riv. crit. Clin. med.*, **68** (Suppl. 6), 1111.
Pollock, T. M.—(1969) *Proc. R. Soc. Med.*, **62**, 1281.
Prehn, R. T.—(1963) *J. natn. Cancer Inst.*, **31**, 791.
Rich, A. R.—(1935) *J. Urol.*, **33**, 215.
Schwartz, E. E., Upton, A. C. and Congdon, C. C.—(1957) *Proc. Soc. exp. Biol. Med.*, **96**, 797.
Schwartz, R. S. and Beldotti, L.—(1965) *Science, N.Y.*, **149**, 1511.
Simonsen, M., Engelbreth-Holm, J., Jensen, E. and Poulsen, H.—(1958) *Ann. N.Y. Acad. Sci.*, **73**, 834.
Sinks, L. F. and Clein, G. P.—(1966) *Br. J. Haemat.*, **12**, 447.
Smithers, D. W.—(1967) *Br. med. J.*, ii, 263 and 337.
Stanley, N. F. and Keast, D.—(1967) *Aust. J. exp. Biol. med. Sci.*, **45**, 517.
Stanley, N. F., Walter, M. N., Leak, P. J. and Keast, D.—(1966) *Proc. Soc. exp. Biol. Med.*, **121**, 90.
Starzl, T. E., Porter, K. A., Andres, G., Halgrimsen, C. G., Hurwitz, R., Giles, G., Terasaki, P. I., Penn, I., Schroter, G. T., Lilly, J., Starkie, S. J. and Putnam, C. W.—(1970) *Ann. Surg.*, **172**, 437.
Steel, C. M. and Hardy, D. A.—(1970) *Lancet*, i, 1322.
Stjernswärd, J.—(1965) *J. natn. Cancer Inst.*, **35**, 885.—(1966) *J. natn. Cancer Inst.*, **36**, 1189.
Strum, S. B., Park, J. K. and Rappaport, H.—(1970) *Cancer, N.Y.*, **26**, 176.
Stutman, O.—(1969) *Science, N.Y.*, **166**, 620.
Trentin, J. J.—(1958) *Fedn Proc. Fedn Am. Socs exp. Biol.*, **17**, 461.
Tyler, A.—(1960) *J. natn. Cancer Inst.*, **25**, 1197.
Von Bernuth, G., Minielly, J. A., Logan, G. B. and Gleich, G. J.—(1970) *Pediatrics, Springfield*, **45**, 792.
Vredevoe, D. and Hays, E. F.—(1969) *Cancer Res.*, **29**, 1685.
Walford, R. L.—(1966) *Science, N.Y.*, **152**, 78.